# Evaluation of plant seed DNA and botanical evidence for potential forensic applications

**DOI:** 10.1080/20961790.2019.1594599

**Published:** 2019-06-10

**Authors:** Cheng-Lung Lee, Yi-Hsin Huang, Ian C. Hsu, Henry C. Lee

**Affiliations:** a Department of Criminal Investigation, Taiwan Police College, Taiwan, China;; b Crime Scene Theater, Qatar Police College, Doha, Qatar;; c Institute of Business and Management, National Chiao Tung University, Taiwan, China;; d Department of Biomedical Engineering and Environmental Sciences, National Tsing Hua University, Taiwan, China;; e Forensic Science Program, University of New Haven, West Haven, CT, USA

**Keywords:** Forensic sciences, forensic botany, tomato seed, DNA extraction, crime scene, non-human evidence, AFLP, STR

## Abstract

Seeds, the reproductive organs of plants, are common as trace evidence from crime scenes. Seed evidence could be grouped into several categories based on the types of crimes they are associated with, including child abuse, homicides and drugs. Most commonly, seeds are examined microscopically and identified to the plant species level to show a linkage between persons and places. More recently, forensic researchers have evaluated the potential for extracting and typing DNA from seeds to further individualize the samples. As a model system, tomato seeds were examined microscopically after different cooking treatments and assessed for the potential to DNA type seeds for variety identification. A sufficient quantity and quality of DNA were recovered from uncooked, digested and undigested tomato seeds for amplified fragment length polymorphism (AFLP) analysis; however, any form of cooking destroyed the seed DNA. A simple microscopic analysis was able to distinguish between a cooked tomato seed versus an uncooked seed. This article is intended to provide an overview of case examples and current techniques for the forensic examination of seeds as plant-derived evidence.

## Introduction

The purpose of this study is to find the relationship between the microscopic exterior comparison and the molecular biological identification method that will be able to quickly screen tomato seed and recover its DNA for forensic application. Our previous study has selected a single fresh or undigested tomato seed to extract DNA and using amplified fragment length polymorphism (AFLP) method to identify for forensic purpose [1].

Various types of plant evidence could be found at the crime scene, some may be on weapons or on clothing due to vomiting, defecation or contents in the autopsied digestive tract. If we could extract DNA from undigested seeds or other plant materials, they could provide valuable evidence during criminal investigations. Currently, only a few published results illustrated how these plant materials can be used for the forensic purpose. Most of these reports were based on microscopic observations which are useful if it is easy to identify the seeds by morphology. To further explore the use of plant evidence in forensic investigations, seeds can only be identified by DNA methods. Cataloging genetic diversity in any species can be accomplished using different morphological or DNA marker systems (short tandem repeats (STRs), AFLPs, single nucleotide polymorphisms (SNPs)). The choice of which molecular marker system to use will depend on the genetic history of the plant species. In any choice of a DNA marker system, it is critical to select markers that will be variable enough in a population to distinguish between individuals even if they are close relatives. Many plants are highly inbred or are back-crossed to a parent line which means that the resultant seeds may share a high percentage of common DNA. In most forensic applications, the basic features and morphology of seeds are examined to try and determine what type of plant species they may have originated from to further demonstrate a linkage between persons and geographic locations. Recently, methods for individualizing plants by DNA have been described [2]. In this article, we provide a novel method to judge whether we could extract DNA from cooked/digested tomato seeds which have not done previously. In below cases where seed evidence has been used, it was other methods as microscopic comparisons. We also set up a model system, and tomato seeds were examined microscopically after different cooking treatments and assessed for the potential to DNA type seeds for variety identification. This article is intended to provide an overview of case examples and current techniques for the forensic examination of seeds as plant-derived evidence. The ability to microscopically identify not just the plant genus and species but also the plant variety may be valuable for establishing linkages between the last meal and a location, for example. When variety identification is not possible using a microscopic method, then extracting DNA from seeds and developing DNA profiles may be a good alternative approach. This study was designed to determine whether the DNA of the commercial and home-cooked tomato seed could be recovered, and whether there are morphological differences between fresh, commercial and home-cooked tomato seeds.

### Seed morphology

As a result of sexual reproduction in plants, seeds with their own unique genetics are formed. The seed includes three regions: the embryo, nutrition source and seed coat [3]. The embryo is the young sporophyte plant that will grow into the full adult plant. Surrounding the embryo is the nutrition source which commonly has starch and other substances to feed the embryo during early growth before the embryo establishes a root and leaf system to support its own growth. Around the whole seed is a layer termed the seed coat. When growth conditions are supportive (i.e. sufficient water and light exist), the seed coat may trigger the germination or sprouting of the seed. The seed develops from special structures in a plant called ovule. The ovule itself is an immature seed, which does not yet contain a viable embryo. It is only when the egg cell inside the ovule is fertilized by sperm cells from pollen that the ovule is called a seed. The ovule is surrounded by integument tissues which produce the seed coat; in some seed plants another layer, the cupule, enclosed the seed. The importance for genetics is that the plant's non-reproductive structures (e.g. leaves, roots), and the cupule, seed coat and integument tissues may have different genetics than the newly formed embryo. This information is critical when deciding what tissues to select for DNA testing.

### The application of seed DNA in forensic science

A forensic scientist’s most important work is to find a piece of potential evidence for crime scene investigation and solving a crime. Ingested food is one of the important roles of forensic evidence obtained during crime scene investigation or medicolegal autopsy. Many materials containing seeds pass through the human digestive system and are still recognizable, thus they can be valuable for providing investigative leads.

One example of how seeds from stomach contents can play an important role in toxicology is described in the following study: poppy seeds from seven different origins (Dutch, Australian, Hungarian, Spanish, Czech, and two Turkish) were analyzed for opiate content [4]. Four grams of each kind of seeds, equivalent to the amount of seeds on two bagels, were ingested by volunteers. For the next 24 h, urine samples were obtained and screened for the presence of morphine and codeine. Poppy seeds from different geographic origins were found to contain a wide variation of morphine (2–251 mg/g) and codeine (0.4–57.1 mg/g) content. Results showed that several kinds of poppy seeds can give positive testing results but after 24 h post-ingestion, all testing results became negative. A significant amount of variation in opiate excretion exists from person to person [4].

As another example of the usefulness of vegetable or fruit seeds recovered from stomach contents, tomato seeds were ingested, both before cooking and after a variety of cooking treatments and then chemically extracted for DNA. Our previous work has shown that the average yield (from three DNA extractions) from a single tomato seed (one seed per extraction) that was dissected into two parts was 100 ng and 62.5 ng for exterior seed coat and interior embryo, respectively, and the digested seed DNA yield was approximately equivalent to the fresh seed [1]. High-quality DNA (40 ng total) could be extracted from only half a seed embryo; a minimal sample input of approximately 1 000 ng of fresh starting tissue weight [1,2]. AFLP analysis is useful for the identification of tomato. The goal of this work by the authors was to determine the effect of different pre-treatments of seeds before ingestion on the ability to perform later DNA testing. In addition, we wanted to evaluate the ability to microscopically identify a tomato variety as compared to a DNA-based method to quickly screen tomato seeds for variety identification.

Non-human trace materials, such as plant and animal products, not only provide important information for criminal investigations by linking a victim, suspect, witness or weapon to a crime, also may provide important investigative leads during criminal investigations or aid in finding clandestine graves, establishing time of death, the verification of an alibi, or linking a person to a crime scene [5,6]. For example, in a recent homicide, many grass seeds were found attached to a blanket that was wrapped around a body that could later be correlated at the species level to similar seeds associated with a suspect’s shoes by microscopic analysis (N. Petraco, personal communication) by microscopic analysis. A simple microscopic analysis of seeds from plants has been used successfully in several forensic cases. For example, the location of blueberry seeds on a suspect’s pants has supported a child abuse case [7]. In another example, a rare cypress seed associated with a body offered important information to help police trace and link a body to the suspect’s house as the primary crime scene [7,9]. Yet another case in which forensic botany disproved a fictitious alibi was through the identification of a rare species of grass seed in the cuff of the suspect’s trouser that linked the suspect to the scene of the assault [7,9]. These cases illustrate how seed evidence may be applied in forensic investigations by the simple microscopic identification of the plant species and the subsequent association to a crime scene.

To further extend the potential use of seeds as evidence, DNA typing is possible. For some plant species, STR and SNP markers have been developed and can be used to individualize a plant sample [7,10,11]. The plants where these techniques have been applied in forensics include crop species, *Cannabis sativa*, and some mosses. As an alternative, AFLPs can be used to determine a genetic match between plant samples [12,13].

At crime scenes, an investigator may find various types of tomato evidence such as spaghetti sauce, canned tomatoes, Bloody Mary mix, fresh tomatoes, tomato juice or tomato seeds on weapons or on clothing due to vomiting, defecation or in autopsied stomach contents. For this reason, tomato seeds were used as a model system in this study since tomato (*Lycopersicon esculentum*) is common to many cuisines around the world.

## Materials and methods

### Fresh versus ingested tomato seeds

**Figure 1.**
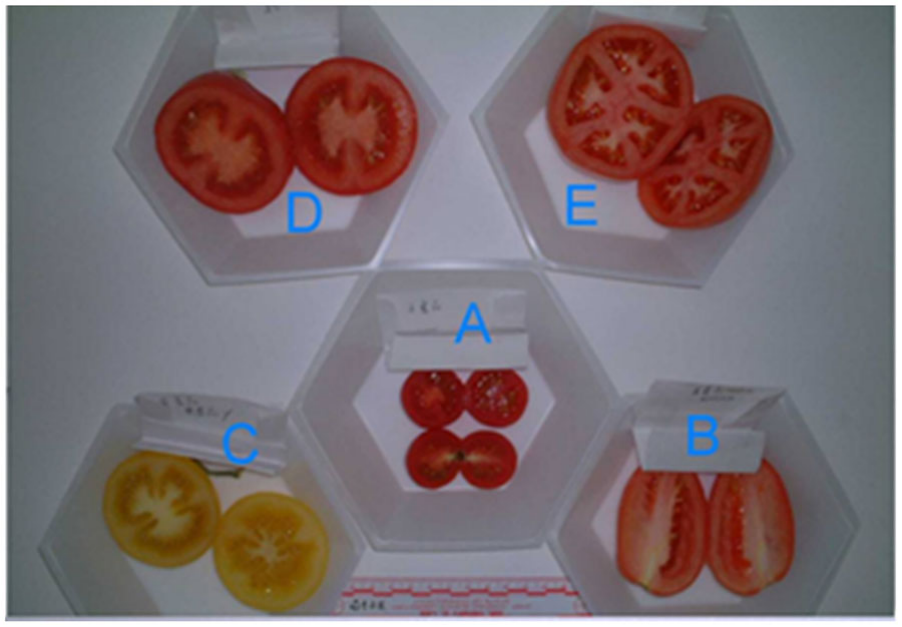
Five cultivars of whole tomatoes (letter means color, diameter; (A) red, 3.5 cm^2^; (B) red, 8.5 cm × 5.4 cm; (C) yellow, 7.0 cm^2^; (D) red, 7.5 cm^2^; (E) red, 9.0 cm × 7.7 cm).

**Figure 2.**
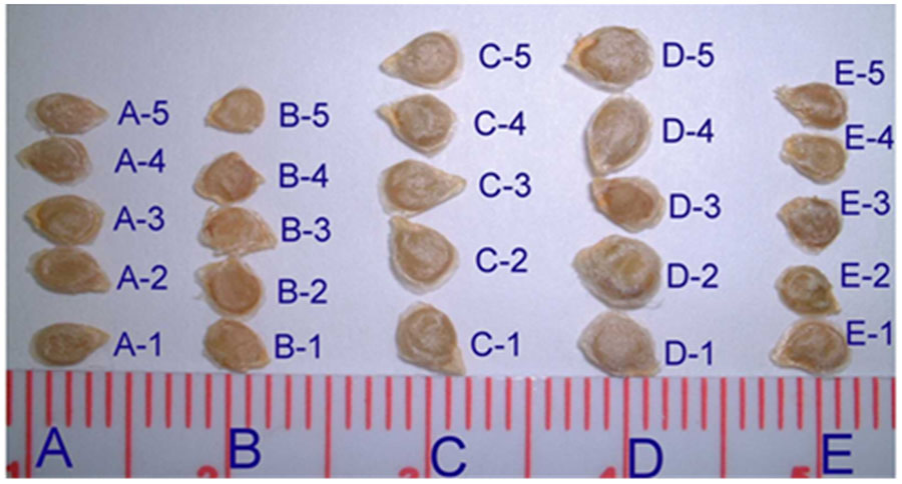
Five cultivars of tomato seeds. It is hard to distinguish different tomato cultivars from seed’s morphological appearance (designated A–E).

As our previous work has shown that tomato seeds were collected from five morphologically different fresh tomatoes obtained from local grocery stores [1,2]. Figures 1 and 2 illustrate five cultivars of tomato seeds. The diameter of the tomato did not correlate to seed size and seed morphological appearance was not a predictor of the tomato cultivar. All they look alike and could not distinguish the difference, thus, we need to develop a new method to distinguish between species and cultivar identification. Approximately 10–25 h after each volunteer ingested a different tomato, excrement was collected. The seed samples were sieved and washed with 10% sodium hypochlorite. After air drying, seeds were examined under an Olympus microscope (model BH-2) at 40× magnification before dissection to avoid other adherent material that might later affect DNA quantity since estimates made from visual comparisons to DNA standards on an agarose gel after electrophoresis are not species-specific.

### Commercial tomato products

Twelve different brands of commercial tomato products were used in this study. There were five kinds of canned tomatoes including “Hunt’s tomatoes petites diced”, “Weis quality diced tomatoes”, “Big Top tomatoes”, “Del Monte Quality diced tomatoes” and “Contadina Roma Style tomatoes”. Seven kinds of tomato spaghetti sauces were used including “Prego pasta sauce”, “Newman’s Own Marinara pasta sauce”, “Health Choice Traditional Pasta sauce”, “Francesco Rinaldi tomato & basil”, “Barilla Basilico tomato & basil”, “Classico traditional favorites tomato& basil” and “DelGrosso tomato basil spaghetti sauce”. Nine of the twelve above listed commercial tomato products contained seeds. Seeds were separated manually from other tomato materials in the can, then collected by washing through a sieve. DNA extractions were carried out for both separated tomato materials (flesh, tissue) and the seed.

### Home-cooked tomato products

Home-made tomato products were prepared by cooking fresh tomato with three methods for different time periods: (i) Fresh tomatoes were boiled at 100 °C for 1, 5, 10, 15, 20, 30 s; 1, 3, 5, 10, 20, 30 min; or 1, 2, 3 h. (ii) Baked tomatoes were prepared in the oven at 218 °C for 10, 20 or 30 min. (iii) Pan-fried tomatoes were prepared at above 100 °C for approximately 3 min. Tomato seeds were collected after each of the cooking treatments and at the specified time intervals.

### DNA extraction

DNA extractions were performed as recommended by the manufacturer’s protocol unless otherwise stated (DNeasy plant mini kit; QIAGEN Inc., Valencia, CA, USA). Seed preparation method No. 1: mechanical grinding of samples in the presence of liquid nitrogen was performed using the Mixer Mill MM300 (QIAGEN Inc.; Retsch Technologies GmbH, Haan, Germany) following the manufacturer’s protocol [7]. Seed preparation method No. 2: a seed was ground with liquid nitrogen to a fine powder by using a 2-mL collection tube and a disposable micropestle (Bel-Art Products, Pequannock, NJ, USA) or a ceramic mortar and pestle (“hand-grinding method”). For the final step, 50 µL of preheated (65 °C) buffer AE was added onto the DNeasy membrane to elute the extracted plant DNA. DNA yields were estimated by comparison with genomic DNA standards (K562, Gibco BRL) after electrophoresis on 1% agarose gels containing ethidium bromide for visualization. A portion of each DNA sample (10 μL) was loaded on the agarose gel for estimating yield. To assess whether the extracted DNA could be amplified for subsequent AFLP analysis [14], a DNA sample extracted from a single representative seed was typed following the manufacturer’s protocol (Plant Mapping kit; Applied Biosystems, Inc., Foster City, CA, USA).

### Comparison of tomato seed morphology

The fresh tomato was used as the positive control in this study. Seeds were manually separated from the tomato tissue and DNA was extracted accordingly. The morphology of the fresh tomato seed and the seed recovered from homemade products and commercial products were examined and compared using an Olympus microscope (model BH-2) at 40× magnification.

## Results

### Fresh versus ingested tomato samples

**Figure 3.**
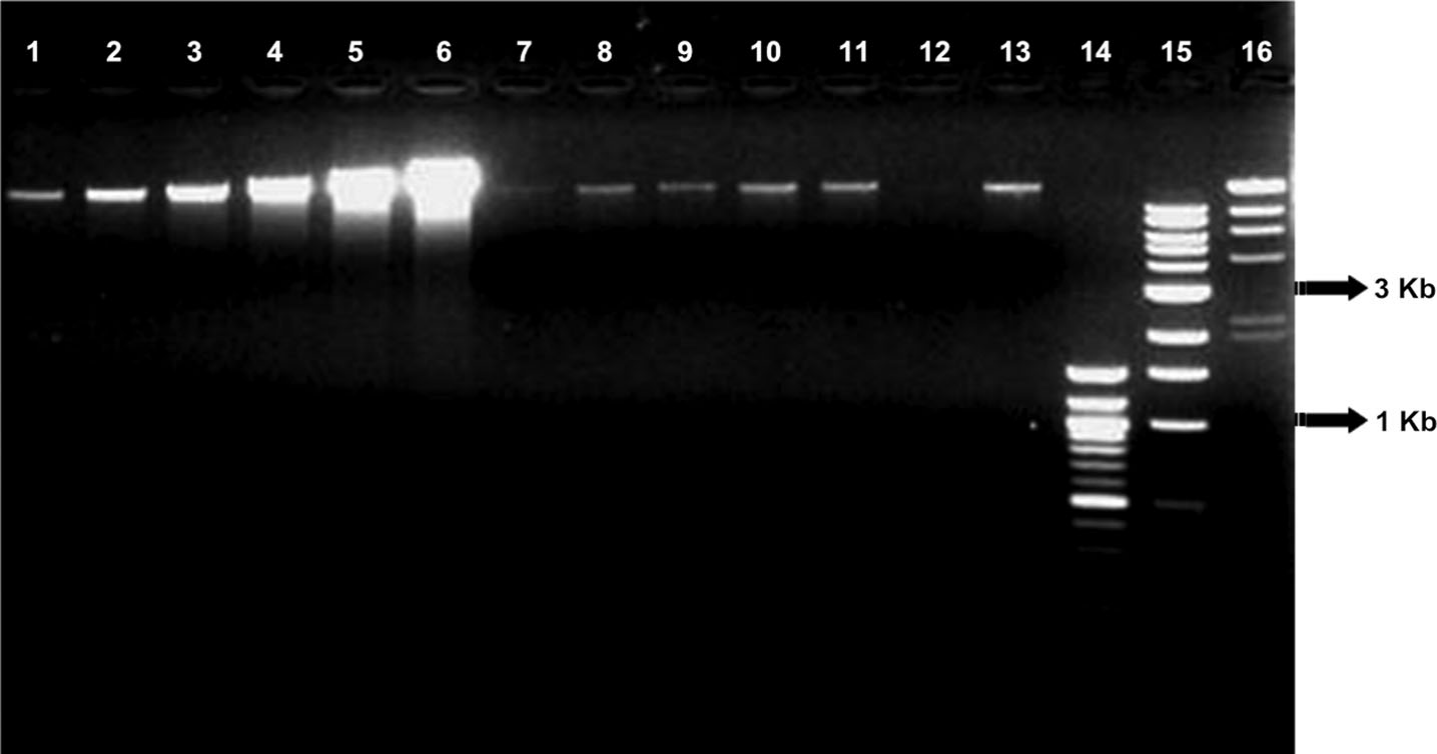
High-quality total genomic DNA could be recovered from dissected embryos (cut in half) and intact tomato seeds (embryo plus seed coat, cut in half) from two of the five tomatoes. Lanes 1–6 are DNA quantitative standards, human K562 (12.5, 25, 50, 100, 200, 400 ng, respectively); lane 7 is half an embryo from tomato 1; lane 8 is half a seed coat from tomato 1; lane 9 is half a seed (embryo + seed coat combined) from tomato 1; lane 10 is half a seed (embryo + seed coat combined) from tomato 2; lane 11 is half a seed coat from tomato 2; lane 12 is half an embryo from tomato 2; lane 13 is an intact seed from tomato 3; lanes 14–16 are standard size markers 100 bp, 1 Kb, and Lambda DNA-Hind III ladder, respectively (New England Biolabs, Beverly, MA, USA). In all cases, the recovered DNA from the tomato seeds or dissected seed components was approximately 12.5 ng/10 μL or less.

**Figure 4.**
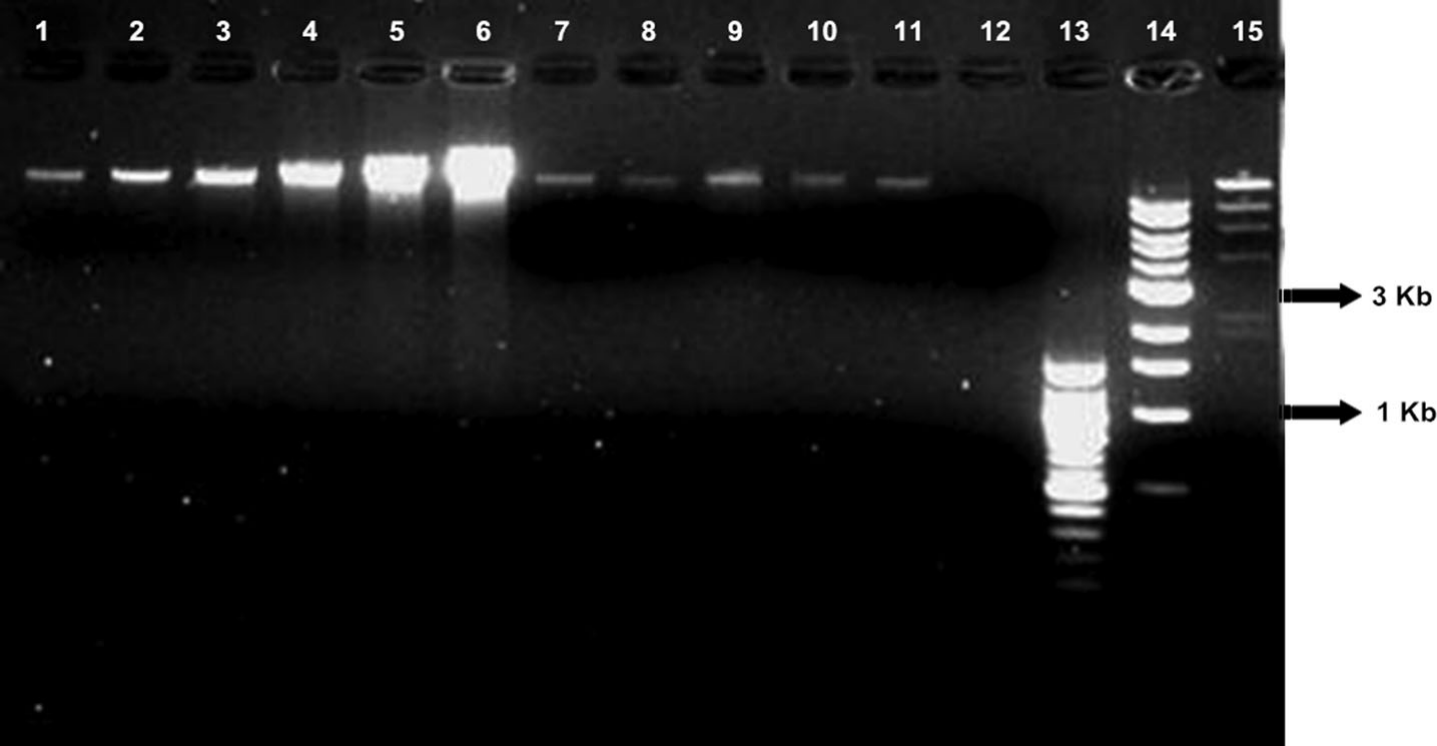
High-quality total genomic DNA was recoverable from a single fresh seed and single fresh tomato digested seed. This DNA was of sufficient PCR quality as tested by the AFLP method. Lanes 1–6 are DNA quantitative standards, human K562 (12.5, 25, 50, 100, 200, 400 ng, respectively) ; lanes 7–10 are single intact fresh tomato seeds 1–4 from one tomato type; lane 11 is from a single, fresh digested tomato seed; lane 12 is a negative control; lanes 13–15 are standard size markers 100 bp, 1 Kb and Lambda DNA-Hind III ladder, respectively (New England Biolabs, Beverly, MA, USA).

The average yield was estimated (from three DNA extractions) from a single tomato seed (one seed per extraction) that was dissected into two parts; the DNA yield was 100 ng and 62.5 ng for exterior seed coat and interior embryo, respectively (Figure 3), and the digested seed DNA yield was approximately equivalent to the fresh seed (Figure 4). High-quality DNA (40 ng total) could be extracted from only half a seed embryo; a minimal sample input of approximately 1 000 ng of fresh starting tissue weight.

To increase DNA yield, hand-grinding of seeds in liquid nitrogen to disrupt cells with a sterile disposable micropestle to release the DNA is recommended. The results indicate that hand-grinding of samples in liquid nitrogen yielded better quality DNA and a greater quantity than with the Mixer Mill. This result was unexpected since the Mixer Mill should apply more force to the cells and yield more DNA. This is probably due to the tube design used for the Mixer Mill and small sized tomato seed samples [7].

**Figure 5.**
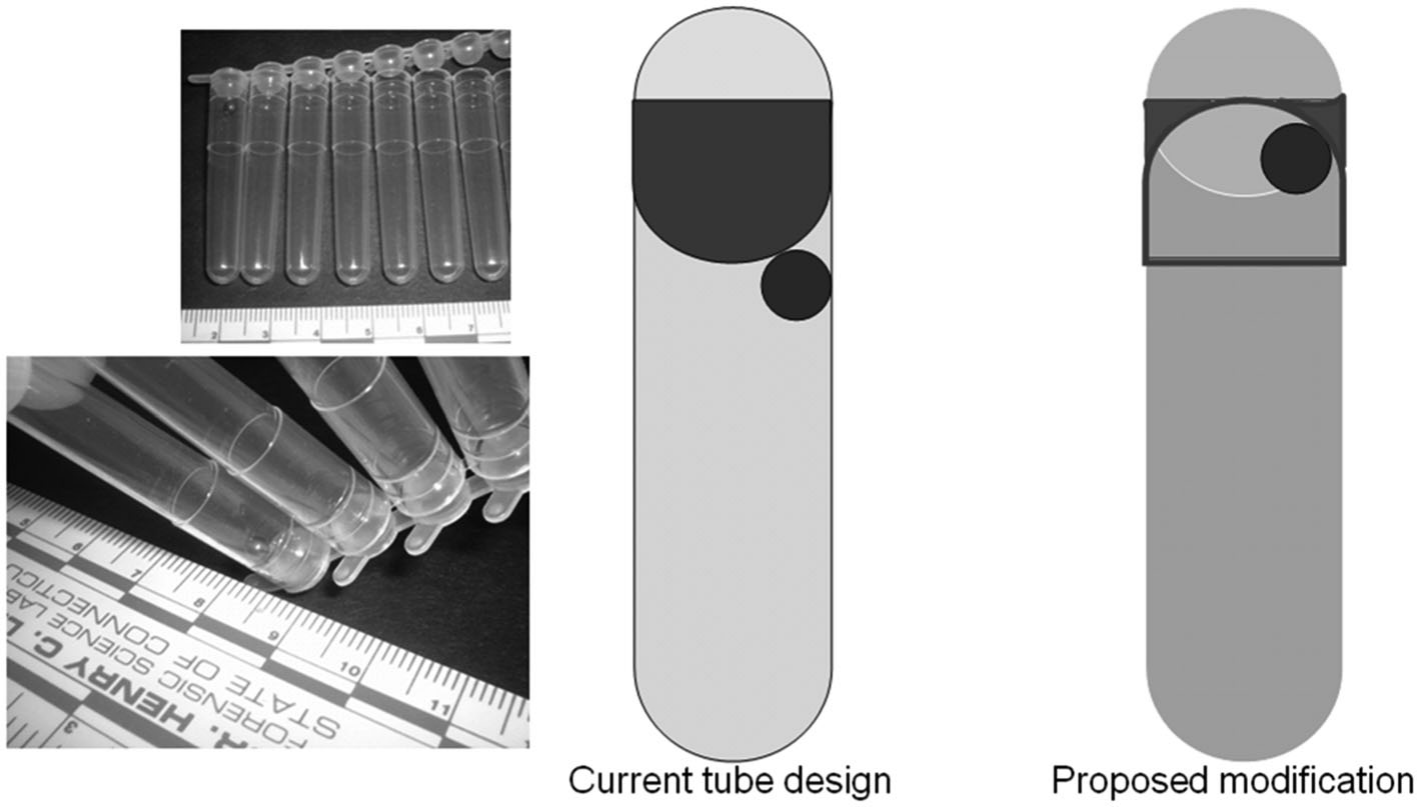
The suggested improvement in tube design may increase single seed DNA yields with the Mixer Mill machine seed preparation method. The cap design is inverted to avoid the space where the seed can become caught between the cap and the tube wall and would result in better tissue disruption and increased DNA yields.

PCR-quality plant DNA was extracted from both exterior seed coat and interior seed embryo using QIAGEN plant DNeasy kit. Fresh tomato seeds, after passing through the human digestive system, also yielded a sufficient amount of DNA for further PCR-based experiments (Figure 4). The main reason for the yield difference between these two procedures was probably due to the sample tube design (Figure 5) of the Mixer Mill. With larger samples (e.g. leaf tissue), the tubes are efficiently allowed for optimal contact between the tissue and the beads used for tissue disruption; however, with significantly smaller tissue samples of a few millimeters in length (e.g. tomato seeds), the samples become trapped in the space between the sidewall of the sample tube and the bottom of the tube cap. This design limits the contact between beads and tissue sample and reduces the efficiency of the grinding procedure, resulting in low DNA yields. A different tube design may improve single seed DNA yields with the Mixer Mill method.

### Commercial and home-cooked tomato samples

**Figure 6.**
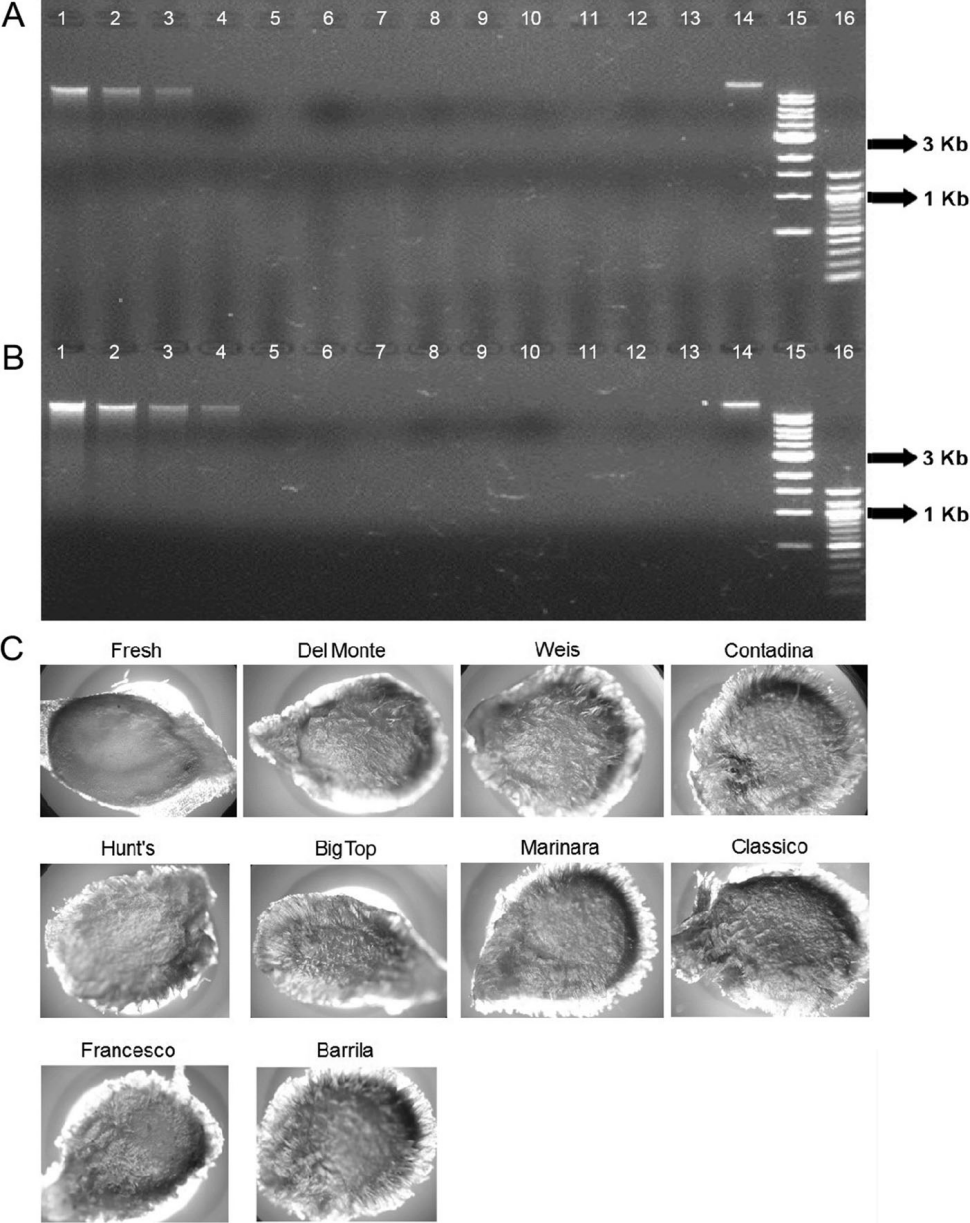
(A) DNA test results of nine seeds from commercial tomato products. Lanes 1–3 are quantitation standards, K562 (50, 25, 12.5 ng, respectively) ; lane 4 is a negative control; lanes 5–9 are DNA extracted from seeds of five canned tomatoes; lanes 10–13 are DNA extracted from seeds of four brands of tomato spaghetti sauces; lane 14 is a DNA extracted from fresh seed; lanes 15–16 are standard size markers, 1 Kb, and 100 bp ladder, respectively (New England Biolabs, Beverly, MA, USA). (B) DNA extracted from 1 mL of tomato sauce and fresh tomato juice. Lanes 1–4 are quantitative standards, K562 (100, 50, 25, 12.5 ng, respectively); lane 5 is a negative control; lanes 6–7 are DNA extracted from two types of canned tomatoes; lanes 8–13 are DNA extracted from six brands of tomato spaghetti sauces; lane 14 is a DNA extracted from fresh tomato tissue; lanes 15–16 are standard size markers 1 Kb and 100 bp ladder, respectively (New England Biolabs). (C) Photomicrographs of the seed surface morphology of nine brands of commercial tomato products and one fresh tomato seed.

**Figure 7.**
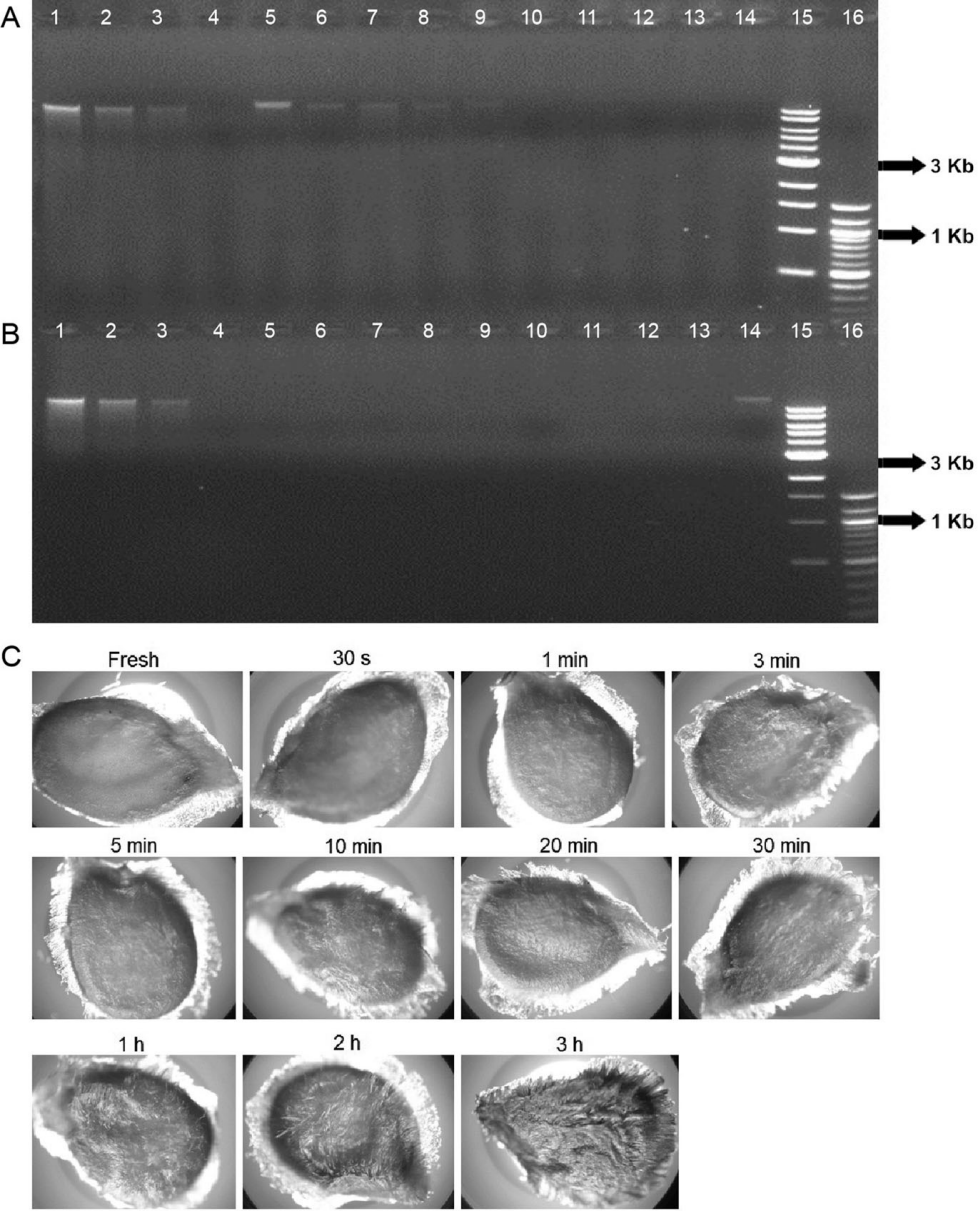
(A) DNA test results from 10 different cooked conditions. Lanes 1–3 are quantitative standards, K562 (50, 25, 12.5 ng); lane 4 is a negative control; lanes 5–14 are tomato seeds that were boiled for 1, 3, 5, 10, 15, 20, 30 s and 1, 3, 5 min; lanes 15–16 are standard size markers 1 Kb and 100 bp ladder (New England Biolabs, Beverly, MA, USA). (B) DNA test results from 10 different cooked conditions. Lanes 1–3 are quantitative standards, K562 (50, 25, 12.5 ng); lanes 4–9 are tomato seeds that were boiled for 10, 20, 30 min and 1, 2, 3 h; lane 10 is a pan-fried tomato seed that was cooked above 100 °C for 3 min; lanes 11–13 are tomato seeds that were cooked at 218 °C for 10, 20 or 30 min); lane 14 is DNA from fresh seed; lanes 15–16 are standard size markers 1 Kb and 100 bp ladder (New England Biolabs). (C) Photomicrographs of the seed surface morphology from seeds cooked at 10 different cooking times (from 30 s to 3 h) at 100 °C and one fresh tomato seed.

Seeds were recovered from all five brands of canned tomatoes and four of the seven brands of tomato sauce. However, none of our DNA extraction protocols produced a detectable amount of DNA from these commercial tomato samples. Figure 6(A, B) demonstrates the DNA extraction yield from a single seed of nine different commercial products. Additional seeds were extracted and the results were consistent with those from the single seed study. DNA was extracted from 1 mL of tomato material from all 12 commercial tomato products. No detectable amount of DNA was recovered from any of those samples (data not shown). Figure 7(A) also shows the DNA extracted from all of the home-cooked tomato samples. Our results indicate that DNA could not be recovered from tomato seeds that had been cooked in boiling water for more than 20 s, and no detectable amount of DNA was obtained from oven baked or pan-fried tomato samples.

### Microscopic examination of tomato seeds

**Figure 8.**
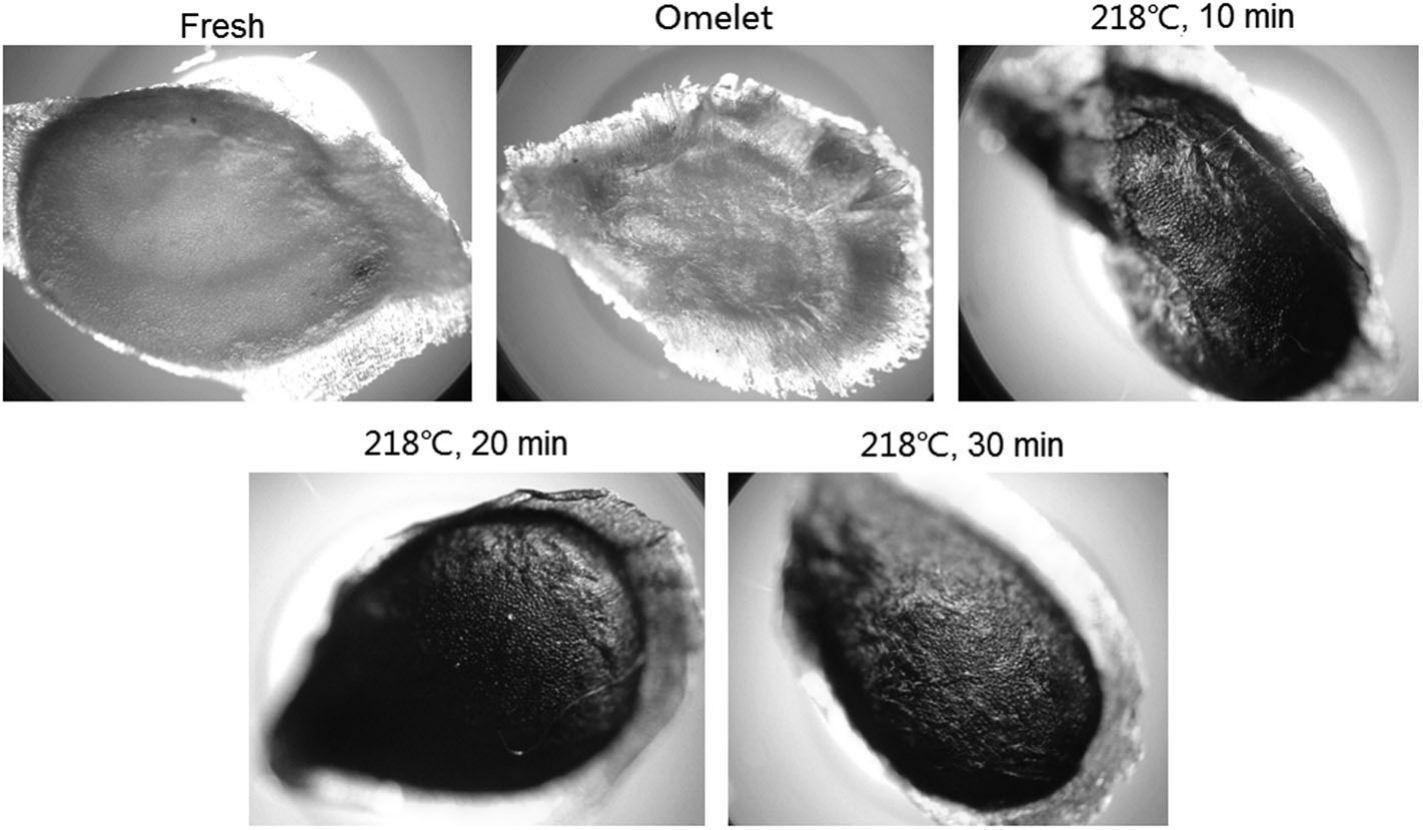
Photomicrographs of the seed surface morphology of fresh and cooked (pan-fried and baked) seeds.

Figures 6(C), 7(C) and 8 show the surface characteristics of tomato seeds under microscopic examination. In fresh tomato seeds, a smooth surface was observed. However, after the seed was cooked for sauces or an omelet, the epidermal hairs on the seed surface became more obvious. A comparative study was conducted by examining tomato seeds boiled for different lengths of time. Epidermal hairs were observed after the seed was boiled for 1 min. After 3 min of boiling, the surface hairs became quite evident (Figure 7(C)). These types of hairs were also observed on the seeds recovered from commercial products. Figure 6(C) shows the seed surface morphology of many different commercial tomato products. The colour of tomato seed darkens with oven baked samples and the seed lost its epidermal hairs after baking (Figure 8).

## Discussion

Our results show that a sufficient quantity and good quality DNA could be extracted from a fresh seed embryo or seed coat; however, DNA could not be extracted from an entire seed of a canned tomato. To resolve this concern, we further found that after high-temperature treatment, the DNA of most tomato seeds will be destroyed. A microscopic examination could be useful to quickly screen tomato seeds to identify characteristic features of a tomato seed’s surface to further distinguish between fresh and cooked tomato samples. The fresh seed had a smooth appearance and the cooked seed had obvious epidermal hairs. This discovery could be used to screen valuable seed quickly for DNA value in forensic casework. At present, there are genetic improvement problems in plant varieties. We shall be very careful to concern this issue in the future application.

The sample collection and processing of seeds are extremely important due to the following reasons: (i) ingested seeds may acquire surface contaminants such as bacteria when they pass through the digestive system; (ii) seed components have different genetic inheritance patterns and (iii) DNA yield from small tissue sources can be limited or insufficient for further testing. Seeds should be cleaned with 10% sodium hypochlorite and water to remove any surface mold or bacteria prior to DNA extraction. Since some DNA-based identification methods are not species-specific during the DNA amplification steps, poor sample preparation may result in complex mixtures which may increase the difficulty of data interpretation. In addition, seed processing needs to be considered when designing the experiment because different parts of the seed will yield different genetic patterns. Tomato seed is formed following fertilization between the plant ovule and a pollen grain. It consists of a seed coat, embryo and storage tissue. The outer layer of the seed develops from the integuments of the ovule. The seed coat is maternal in origin whereas the embryo is a unique (F1) hybrid of parental genetics due to recombination events and independent assortment. Whenever possible, it is preferable to dissect the embryo (internal portion of the seed) prior to DNA extraction to avoid the maternal component of the seed coat or any possible surface contaminants. Finally, since seeds are often small, sample quantity may be limited and a sufficient amount of DNA recovery is necessary for further testing. Tomato seeds serve as a proof in principle for establishing the amount of DNA one might expect to recover from smaller sized single seeds.

The plant DNeasy kit from QIAGEN was used to efficiently process intact seeds and dissected embryos to obtain PCR-quality DNA. The use of forensic botanical evidence from crime scenes for DNA typing is still in the developmental stages for difficult samples such as plant matter from stomach contents and excrement [1,2,7]. In our study, we extracted and analyzed tomato seed’s DNA from commercial products such as spaghetti sauce, tomato sauce and canned tomato. We found that none of our DNA extraction protocols produced satisfactory yields for quantity and quality of DNA when using commercial tomato seeds as starting materials. Our results suggest that the DNA yield is directly related to the temperature and/or pressure of how the tomato products were processed.

In addition, a novel procedure was developed to combine the microscopic analysis of seed morphology and the yield determination of DNA from the seed. This procedure could be used to distinguish whether a tomato seed originated from fresh tomato, homemade tomato sauce or a commercial product and if it is likely to yield a DNA profile. While the microscopic identification of a plant seed to the species level may be sufficient for analyzing some forensic cases, many other types of seeds will require molecular biological (DNA) identification either because they are not morphologically distinguishable as intact seeds or because fragmentation has limited any identifiable features for further characterization. This study was designed to further define the criteria for choosing to process seed evidence for a DNA profile for identification and individualization.
